# Experiences of members of the care triad on transitioning from home to a nursing home: a qualitative study

**DOI:** 10.1186/s12877-025-06586-1

**Published:** 2025-11-04

**Authors:** Stefanie Skudlik, Katharina Lüftl, Regina Thalhammer, Melina Zaglacher, Martin Müller

**Affiliations:** 1 Faculty of Applied Health and Social Sciences, Rosenheim Technical University of Applied Sciences, Hochschulstraße 1, Rosenheim, 83024 Germany; 2 Department for Primary Care and Health Services Research, Nursing Science and Interprofessional Care, Medical Faculty Heidelberg, Heidelberg University, Heidelberg, Germany

**Keywords:** Transitional care, Qualitative study, Older adults, Nursing home, Informal caregivers

## Abstract

**Background:**

The transition from home to a nursing home is an emotionally and organisationally challenging process for the care triad, consisting of older adults in need of care, their informal caregivers, and health professionals. Although this transition is known to occur in three phases, no study has comprehensively examined the experiences of care triad members across all phases, and evidence-based interventions to support informal caregivers and older adults through this highly stressful process are lacking. This study aimed to explore the experiences of care triad members, with a focus on identifying problems, strategies, required support, and suggestions for improvement across all three transition phases. These insights will serve as a basis for developing an intervention to enhance the transition.

**Methods:**

We conducted a qualitative study using semi-structured interviews with participants representing the care triad in Germany. The interview guides were informed by a transitional model and prior literature. Data were analysed using qualitative content analysis.

**Results:**

A total of 29 interviews were conducted. Key problems included limited care options, complex and often unsuccessful decision-making processes, transitional gaps, and a reduction in both autonomy and participation for older adults. Healthcare professionals showed limited and inconsistent involvement in decision-making and providing support. Informal caregivers often used indirect communication to avoid the sensitive topic of nursing home admission, aiming to prevent conflicts. Essential support needs were collaborative decision-making, adequate preparation time, and emotional support. Recommendations emphasized the importance of initiating care discussions early in the transition process.

**Conclusion:**

Transitions from home to nursing homes are often delayed due to postponed decisions, family disagreements, and a lack of support from healthcare professionals. Informal caregivers frequently shoulder the burden, feeling overwhelmed and unsupported. As a result, transitions are often rushed, leading to a loss of autonomy and identity among older adults. Proactive planning and counselling could help prevent these negative outcomes. Interventions should focus on encouraging the early engagement of older adults and informal caregivers in planning their care situation, with consistent professional support throughout the process to ensure their participation and autonomy are maintained.

**Supplementary Information:**

The online version contains supplementary material available at 10.1186/s12877-025-06586-1.

## Introduction

As populations age and the prevalence of complex care needs increases, long-term care transitions have become a pressing concern for health and social care systems [1, 2]. Many older adults prefer to stay in their own homes for as long as possible. This wish is often linked to the desire for independence, routine, and social engagement [3]. However, when care needs exceed the capacities of informal caregivers despite involving home care services (HCS), admission to a nursing home (NH) may become necessary [2]. The transition to a NH is not merely a logistical change but also an emotional and social process [2, 4, 5]. It affects older adults in need of care, their informal caregivers, and healthcare professionals (HPs), who together form what is often referred to as the “care triad” [6, 7]. Each member of this triad experiences the transition differently, shaped by their roles and responsibilities. To design effective interventions for improving the transition process, it is therefore essential to gain insight into these experiences.

## Background

### Conceptualising transitions to nursing homes

Transitions from home to a NH have been conceptualised as a multi-phase process, typically encompassing the pre-transition phase (initiation of discussions and decision-making), the mid-transition phase (preparation and relocation), and the post-transition phase (adjustment to the new living environment) [8]. The TRANSCIT model (Transition, Support, Communication, Information, and Time) provides a comprehensive framework for understanding the needs of informal caregivers and older adults in need of care during this process. It emphasizes the importance of sustained partnership between older adults in need of care, informal caregivers, and HPs across the transition trajectory. This partnership relies on clear communication, tailored support, good information, and enough time to prepare and adapt [8].

### Challenges and emotional impact of transitions to nursing homes

This need for partnership becomes especially critical in light of the emotional burden and significant challenges often associated with transitions from home to a NH [2, 4, 5]. In practice, transitions often fall short of this ideal: communication breaks down, support is fragmented, and the needs of older adults and their informal caregivers are frequently overlooked [9, 10]. These shortcomings have significant consequences for those involved: Transitions often disrupt everyday routines, alter established roles, and weaken long-standing social ties within the care triad [9–12]. In addition, NHs are frequently associated with loss of autonomy, social isolation, or even the final stage of life. These negative associations can intensify resistance to the transition and create emotional distress for both older adults and their informal caregivers [13]. Also, adapting to life in a NH is rarely straightforward. It is shaped by a range of factors, including age, health status, social connectedness, relocation circumstances, and institutional characteristics [14, 15]. Research shows that older adults with planned transitions adjust more quickly than those with unplanned transitions, who often struggle with the sudden loss of their familiar environment and face difficulties navigating the new setting [16].

### Research gaps and limitations of current studies

These findings highlight the importance of structured, well-prepared transitions. Yet despite extensive international research on the transition to NHs, there is still limited insight into how all members of the care triad experience this process — particularly in the German healthcare context [17]. To our knowledge no study has comprehensively examined the experiences of all care triad members across the transition process using a distinct transition model. Most existing research focuses on individual actors — for instance, informal caregivers of people with dementia — or targets single phases, typically the post-transition phase. As a consequence, existing interventions often lack a comprehensive perspective. They frequently fail to provide support across all phases of the transition and rarely include the experiences of older adults themselves as part of the care triad [17–20]. Furthermore, the transition from home to NH has received less attention as the transition from hospitals to NH [8]. Thus, more knowledge about how members of the care triad experience such transitions is needed in order to inform the development of comprehensive interventions.

## Methods

### Aim

**Fig. 1:**
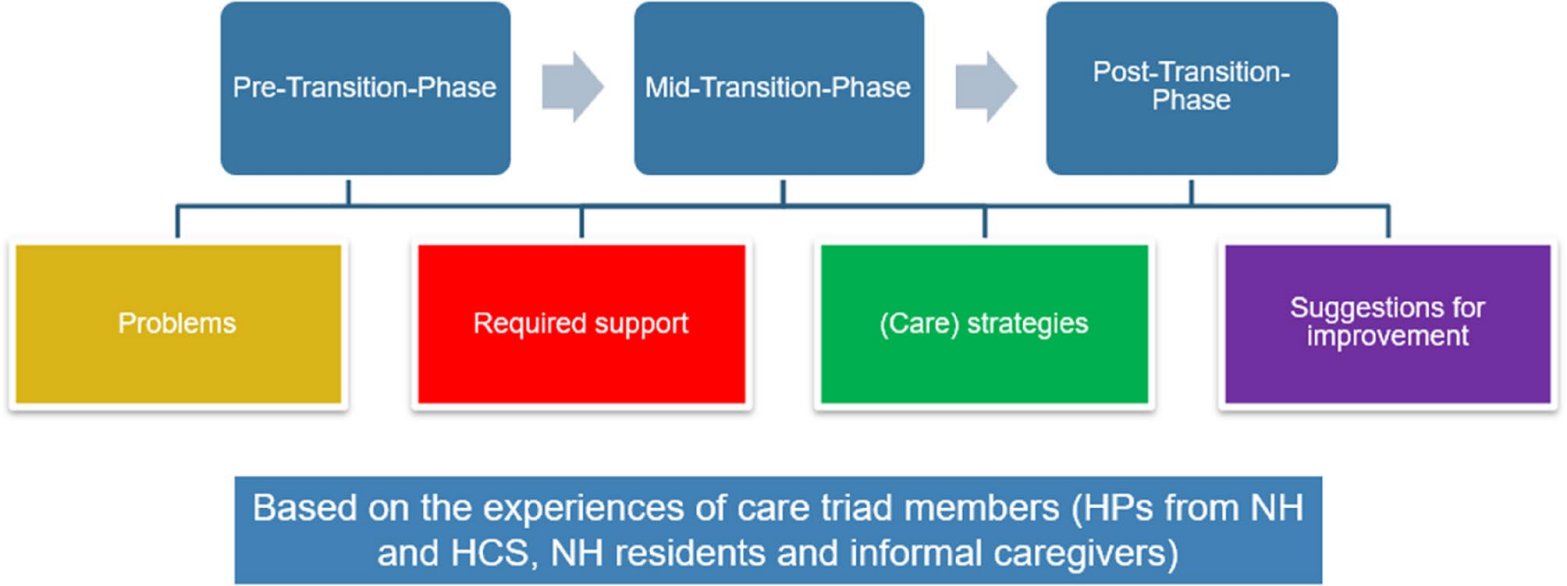
Research questions

The aim of this study was to explore the experiences of care triad members (HPs from NHs and HCS, NH residents, and informal caregivers) across the three phases of the transition from home to NH. Specifically, we analysed their experiences regarding four themes: problems, required support, (care) strategies, and suggestions for improvement (Fig. 1). These themes emerged from our scoping review [17].

The insights are intended to inform the development of a targeted intervention as part of a broader project, which also includes a scoping review [17] and an expert workshop (to be published elsewhere).

### Study design

The present study is part of a larger project, which aims to develop and test a complex intervention to improve the transition from home to NH and follows the UK Medical Research Council (MRC) framework for development and evaluation of complex interventions [21, 22]. We opted for a qualitative study using a qualitative descriptive approach [22] to create knowledge that is closely aligned with the empirical data and can be immediately integrated into the further research process. This ensured that the findings could be discussed in a workshop with care triad members, forming a basis for participatory intervention development.

### Participants

#### Setting/context

NHs in Germany vary in size, accommodating from a few dozen to several hundred residents with diverse care needs. Staff typically includes skilled nurses, nursing assistants, and social care assistants, who focus on leisure activities and participation. In Germany, most citizens are covered by a mandatory federal long-term care insurance, but additional payments are often required, either from personal funds or federal social care. NH admission requires an assessment by the Medical Service of the long-term care insurance, classifying care needs into grades 1 to 5. Full-time residential care benefits start from grade 2.

#### Study participants

The participants were HPs from NHs and HCS, NH residents (who have recently experienced a transition), and informal caregivers of NH residents.

HPs were recruited by phone call and email in south-east Bavaria, Germany, from an existing network of cooperation partners, using convenience and snowball sampling due to the ongoing challenges in many facilities caused by the effects of COVID-19. We included HPs with at least one year of practice in the respective setting and a minimum of six months with the same employer. We assumed that such HPs had gained sufficient experience with transitions from home to NH during this time. Additional criteria included significant experience with these transitions, determined by self-evaluation.

NH residents and informal caregivers were recruited through gatekeeper access by NH staff. This approach was selected to meet the ethics committee’s requirement of including only individuals assessed as being emotionally stable and not at risk of psychological distress. Informal caregivers of residents who had lived in the NH for at least six weeks, but less than two years were included, as we assumed that the transition experience would still be relatively recent, yet not too immediate. Similarly, NH residents aged 65 or older who had lived in the NH for at least six weeks but not more than six months and had transitioned from home were included. We did not restrict inclusion to specific diagnoses or cognitive impairments, aiming for a broad picture. However, residents with cognitive impairments or those under legal guardianship were excluded from personal interviews. Instead, we interviewed informal caregivers of individuals with conditions such as dementia to ensure that their experiences were represented. All participants provided written informed consent. We sought to include members of the same care triad, but for feasibility considerations, this was not a mandatory requirement.

### Data collection

Data were collected through semi-structured individual interviews conducted between December 2022 and February 2023. The interview guides were developed based on the TRANSCIT model [8], incorporating its three transition phases and the key elements. The guides included key questions and specific follow-ups. A narrative approach was used for residents to encourage open sharing of experiences, featuring a few guiding questions designed to prompt storytelling. Table 1 presents key questions from interview guide for HPs in NHs, selected as one of the most structured ones. Key questions from other guides can be found in supplementary file 1. The guides were pre-tested with two volunteers, confirming their clarity and comprehensiveness, requiring no changes.

**Table 1:** Key questions from the interview guide for health professionals in nursing homes

Part I Pre-Transition Phase: First, we’ll focus on the decision-making process, just before individuals decide to move into a nursing home.	
1. Tell me, what is the process in your facility when someone inquires about a nursing home placement? 1.1 Key Elements: Information/Support To what extent are you aware of how the decision to move into a nursing home is made by those affected and their families? 1.2 Key Elements: Support/Communication/Time/Information What information do the individuals and their families receive from you when they inquire about a nursing home placement in your facility? And what information do you need from the interested parties? 1.3 Key Elements: Communication/Time From your experience, what feelings do the families and individuals experience during the decision-making phase?	

Part II Mid-Transition Phase: Now, let’s focus on the time period after the decision for the nursing home has been made up until the time of move-in.	
2. What happens after the decision for the nursing home? 2.1 Key Elements: Support/Information/Communication/Time How are the families supported in the preparations for the move to the nursing home? 2.2 Key Elements: Communication/Time In your experience, what feelings do the future residents, and their families have during the transition phase until moving into the nursing home?	

Part III Post-transition Phase : Now, I’d like to discuss the time after the move-in.	
3. What happens after the move in terms of day-to-day life? 3.1 Key Elements: Communication/Time What is particularly important to you in collaborating with the new residents and their families after the move-in? 3.2 Key Elements: Support/Information/Time/Communication To what extent are the families involved in the daily life of the nursing home residents? 3.3 Key Elements: Support/Time How does the further adjustment in the nursing home proceed?	

Part IV Conclusion: Looking back at the entire transition process from decision to move into the nursing home, what is working well in your facility, and what could be improved?	
Open-ended closing question: Is there anything else you would like to add that I haven’t asked about?	

The interviews were conducted by the first author, a registered nurse with advanced degrees and experience in qualitative research in long-term care. Interviews were audio-recorded, transcribed verbatim, and supplemented with field notes. The audio recordings were deleted after transcription and the transcripts were pseudonymized. Only the first author had access to a password-protected file linking the pseudonyms to the real names. NH resident interviews took place in private rooms at their NH, while informal caregivers or HPs could choose between in-person or telephone interviews. The procedure for in-person and telephone interviews was the same, starting with introduction, explaining voluntary participation, data protection, and the interview process, followed by the interview itself. Some interviews included two participants or an additional person, per participants’ requests. The sample size was based on assumed data saturation [23]. Basic personal information was also collected from interview participants (Table 2).

**Table 2:** Personal information collected from participants

Personal information	Informal caregivers	NH residents	HPs from HCS/NH
Name	X	X	X
Gender	X	X	X
Age	X	X	X
Duration/frequency of HCS use before relocation	X	X	
Time elapsed since relocation	X	X	
Qualification			X
Professional position			X
Scope of employment			X
Length of employment (current employer)			X
Total work experience			X

*NH *nursing home,* HP *health professional,* HCS *home care service

### Data analysis

Data were analysed using content-structuring qualitative content analysis [24] using the software MAXQDA 2022 [25] by two researchers (StS and KL). The three transition phases of the TRANSCIT model guided the overall structure of the analysis. However, the key elements listed in the model were not used as deductive categories, as they were considered too narrow to adequately reflect the data. Instead, transcripts were first coded using a category system based on the transition phases as deductively derived main categories, and thematic subcategories such as experienced problems, support needs, care strategies, and suggestions for improvement, which were informed by our previously conducted scoping review. Then, inductive categories were developed, and initial category descriptions and coding rules were created. Codebooks with descriptions and anchor quotes were reviewed and adapted by the research team. The separate category systems for each target group were repeatedly compared and refined. Several reviews and discussion sessions were held by the research team.

### Trustworthiness

To ensure trustworthiness, we followed the first two Glassick Criteria [26] (clear purpose and adequate preparation) by formulating a precise research question and conducting a literature review to identify research gaps. Lincoln and Guba’s criteria [27] (credibility, transferability, dependability, confirmability) guided our research. Credibility was achieved by thoroughly reviewing transcripts and team discussions within the core research team, as well as by presenting and discussing findings with an extended interdisciplinary team, experienced with transitions, to validate interpretations. Codes were assigned by two researchers, reviewed by other authors and refined in team meetings. Transferability was enhanced by including diverse target groups. Dependability was ensured through careful documentation of all research steps in a research diary and meeting protocols. Confirmability was maintained through team meetings to avoid bias. Results were discussed in a workshop with care triad members, contributing to both credibility and transferability. The study adhered the Standards for Reporting Qualitative Research (SRQR) guideline [28] (see supplementary file 2).

## Results

### Participant characteristics

We interviewed HPs from HCS (*n* = 7) and NH (*n* = 6), NH residents (*n* = 8), and informal caregivers of NH residents (*n* = 8) (Table 3). Participants came from five different NHs and HCS, respectively.

HPs from HCS had a median age of 52 years (range: 30–57). HPs from NH had a median age of 58.5 years (range: 56–60). All HPs from both settings were qualified as skilled nurses, mainly comprising women (*n* = 12).

NH residents (*n* = 8) had a median age of 86 years (range: 65–95) and were equally split between men and women. The median time since admission was 8.5 months (range: 1.3–12).

Informal caregivers (*n* = 8) had a median age of 54 years (range: 48–79) and were all women, most of them daughters of the residents (*n* = 4). The median time since the associated resident’s admission was 8.5 months (range: 2–24).

The interviews with informal caregivers lasted from 30 to 70 min, averaging 47 min. Those with NH residents ranged from 30 to 60 min, averaging 33 min. Interviews with HPs lasted from 30 to 60 min, averaging 48 min. After 27 interviews, data saturation was assumed as no new themes emerged, and dense descriptions of the experiences had been collected.

**Table 3:** Participant characteristics

	HPs from HCS (*n* = 7)		HPs from NH (*n* = 6)		NH residents (*n* = 8)		Informal caregivers of NH residents (*n* = 8)	
	**n**	**%**	**n**	**%**	**n**	**%**	**n**	**%**
Gender female male	6^1^ 1	86 14	6 0	100 0	4 4	50 50	8 0	100 0
Qualification skilled nurse	7	100	6	100				
Professional position management (care/social care) nurse	5 2	71 29	5^2^ 1	83 17				
Scope of employment full-time part-time	6 1	86 14	2 4	33 67				
Use of HCS Yes No					4 4	50 50	4^3^ 4	50 50
Relationship to the resident spouse/wife daughter daughter-in-law friend sister							2^3^ 3 1 1 1	25 37,5 12,5 12,5 12,5
	**Median/Range**		**Median/Range**		**Median/Range**		**Median/Range**	
Age (years)	52 (30–57)		58,5 (56–60)		86 (65–95)		54 (48–79)	
Length of employment (years)	9 (5–27)		11 (2–32)					
Total work experience (years)	26 (5–37)		23,5 (19–39)					
Duration of use of HCS (months)					9 (0,7–36)		4 (0,7–12)	
Frequency of use of HCS (times per week)					7 (1–14)		14 (7–21)	
Time elapsed since admission (months)					8,5 (1,3–12)		8,5 (2–24)	

*HPs *health professionals, *HCS *home care service, *NH *nursing home

^1^ *Two interviews were conducted simultaneously with two HPs, as requested by them*

^2^ *One social care manager also serves as a nurse*

^3^ *One informal caregiver experienced the transition to NH of both her mother and husband*

An overview of the categories and the contribution of the different participant groups to each category is given in supplementary file 3. The results section is structured according to the sequence of the three different transition phases. Emotional support, reassurance, and confidence was identified as a subcategory within both “required support“ and “(care) strategies“ in every transition phase and is reported at the end of the category listing.

### Pre-transition phase

**Fig. 2:**
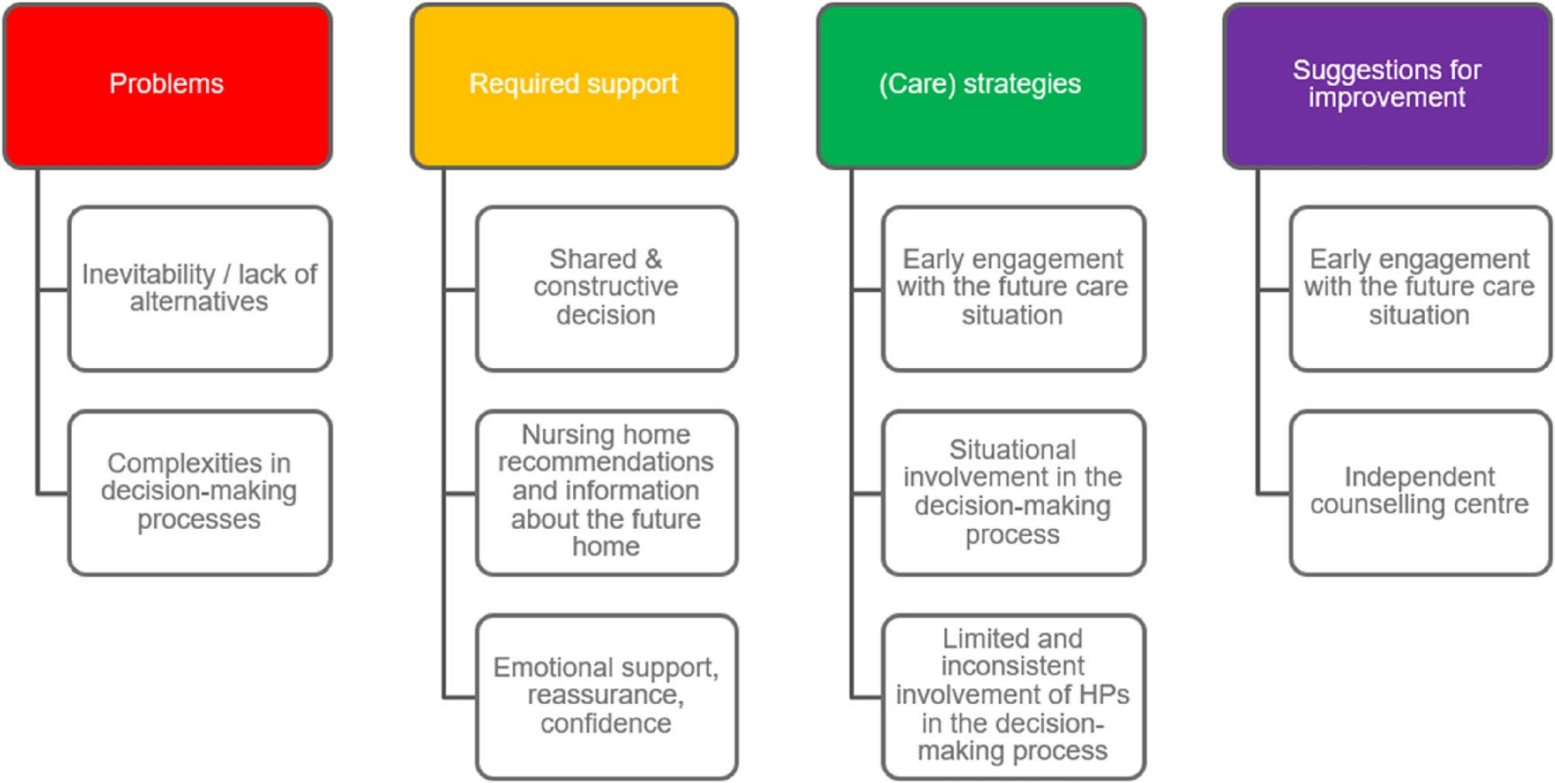
Overview of categories from the Pre-transition phase

Figure 2 provides an overview of the various categories of the pre-transition phase.

#### Pre-transition phase: problems

##### Inevitability/Lack of alternatives

Participants of all groups noted that circumstances such as acute health incidents (e.g., strokes, falls), the lack or loss of informal caregivers, overwhelming burden on informal caregivers, and a decline in health or cognitive capabilities culminate in situations where NH admission became inevitable. This frequently led to abrupt decision-making pressures and hurried transitions. A resident reported that the sudden loss of supportive individuals led to an unplanned admission into a NH:

*“Yes*,* actually*,* I didn’t want to go to a nursing home. […] And then*,* when I was here and my wife died*,* shortly afterwards the women who had been helping my wife and me with the household suddenly cancelled*,* saying they didn’t want to help anymore for reasons I couldn’t understand.”* (NH5_resident2).

An informal caregiver described how her husband’s dementia and frequent night falls, along with other responsibilities, led to the NH placement:

*“And then it happened […]*,* he fell six times at night. […] I couldn’t do it anymore. I was already completely at my limit because when you do that seven days a week and then go to work and have to go shopping and take care of everything else*,* it was too much.”* (NH5_informalcaregiver2).

Even interpersonal conflicts between professional caregivers and persons in need of care can lead to such a sudden need for a transition, as one HCS manager stated:

*“[…] the 24-hour carer couldn’t stand it any longer. […] And she [the person in need for care] was so … not frail*,* but she was very demanding. Interpersonally*,* it was very stressful for carers. […] And no carer came anymore. […] She had to go into short-term care quickly. And now she’s staying.“* (HCS1_HCSmanager1).

##### Complexities in the decision-making processes

All groups described disagreements within families and varied perceptions among the care triad members, along with delays in decision-making and lack of stakeholder involvement and guidance, leading to difficulties in the decision-making process. Informal caregivers reported encountering resistance from care recipients when discussing transitioning to a NH. One caregiver explained why conversations with her father about moving to a NH reached an impasse, despite multiple conversation efforts:

*“That was out of the question for him. […] He fought against it tooth and nail. […] And he absolutely refused. And eventually*,* we gave up.”* (NH5_informalcaregiver1).*“That was out of the question for him. […] He fought against it tooth and nail. […] And he absolutely refused. And eventually*,* we gave up.”* (NH5_informalcaregiver1).

Accusations from the older adult in need of care exacerbate the pressure and guilt on informal caregivers, even when the rest of the family supports the informal caregiver in their decision for the NH placement, as one informal caregiver described:

*“My children said: ‘Mum*,* he has to go in. ' […] I was still so undecided*,* because then he [her husband in need of care] said*,* ‘Yes*,* you send me away’*,* and everything*,* and then I always blamed myself so much.“* (NH2_informal caregiver1). NH residents often acknowledged the inadequacy of home care yet attempted to deny or overlook this. One resident, who had repeatedly fallen at home and knew that she would not be able to maintain her care at home for much longer, explained her rejection of the NH and her postponement of the decision:

*“And so many things like that happened that I thought to myself*,* something has to happen*,* right? I always put it off*,* always put it off. No*,* never in a nursing home*,* never! […] My mum once said: ‘If I had to go into a home*,* I would die in there.’ And yes*,* I thought to myself*,* you’re right.“* (NH1_resident 1).

In contrast, NH and HCS were seldom involved in decision-making processes and HCS were often presented with finalised decisions with contract cancellations:

*“Yes*,* so they [the informal caregivers] call and say: ‘Starting next month*,* Mum will be in a nursing home. She won’t be coming back.‘”* (HCS1_HCSmanager1).

Informal caregivers also indicated that they did not involve HPs in the process. An informal caregiver expressed feeling isolated during the decision-making process and noted a lack of guidance and counselling from HPs:

*“You just can’t seem to get support anywhere. Until you’ve painstakingly pieced together everything you can do and must do yourself.”* (NH5_informalcaregiver1).

#### Pre-transition phase: required support

##### Shared and constructive decision

HPs from NH and HCS and informal caregivers emphasized the necessity for a shared decision-making process involving all stakeholders. If circumstances allow, an attempt is made to reach an agreement, as one NH manager explained:

*“Most residents*,* if they are still cognitively capable and in a position to participate in the decision*,* then*,* of course*,* it is in combination with the resident that this decision [for the NH] has been made. This is also very important to most informal caregivers.“* (NH5_NHmanager1).

A HCS manager also stressed the importance of shared decisions between older adults and their informal caregivers:

*“It’s really important to talk. And they also need that*,* to come to some kind of agreement eventually.”* (HCS4_HCSmanager1).

Informal caregivers’ statements support this. One informal caregiver reported that she would never have been able to make the decision in favour of a NH without her mother’s consent:

*“[…] because I didn’t want to go over her head and say: ‘Mum*,* you go there’ and that’s it.”* (NH3_informalcaregiver1).

Nursing home recommendations and information about the future home.

All groups emphasize the importance of NH recommendations and gathering information. Pre-existing personal connections within the NH are seen as facilitating the transition. One resident mentioned having a friend already in the NH, which eased their decision to relocate there:

*“She [friend of resident] said: ‘If anything happens*,* come and see me in the nursing home. It’s nice*,* the people are nice.‘”* (NH2_resident1).

#### Pre-transition phase: (Care)strategies

##### Early engagement with the future care situation

Participants of all groups stated that some informal caregivers and older adults proactively engaged with the prospect of potential transition to a NH. Some residents described making the decision in collaboration with their family and HPs. One resident recounted how he independently decided to transition into the NH, though with guidance from his general practitioner (GP):

*“I said*,* ‘I’m just alone at home. Can barely manage on my own anymore.’ Then she [his GP] said*,* ‘What would you like to do then?’ I said*,* ‘The best thing would be somewhere like [name of NH] or something.’ She said*,* ‘That’s a reasonable answer and a reasonable thing’. Then she set everything in motion.”* (NH4_resident1).

Another resident expressed that the decision was his sole decision and that he had planned it without outside assistance:

*“I only said to my son*,* ‘Here’s your car key*,* sell your car*,* I’m going to the home.’ Then I drove there [to the NH]. […] I asked if a room was available. […] And then I moved in.”* (NH3_resident3).

HPs reported that decision-making aids, such as short-term care, are frequently employed. They suggested that proactive engagement would greatly facilitate the transition and acclimatization process:

*“Well*,* and then there are some who really consciously engage with it*,* the older adults. […] And that’s when the move-in or the adjustment phase is relatively easy.“* (NH3_NHnurse2).

Informal caregivers also sometimes considered the possibility early on, having discussions with the older adults in need of care and gathering information about potential transition to a NH, as one informal caregiver explained:

*“I have thought about it. Of course*,* I shared it: ‘Mom*,* if it doesn’t work out anymore with home care and so on.’ […] I asked the lady [NH manager] about costs and this and that and whether it would be possible*,* not that it’s necessary now*,* but if.”* (NH3_informalcaregiver1).

##### Limited and inconsistent involvement of health professionals in the decision-making process

HPs from NHs and HCS reported varying degrees of involvement of the HCS in the decision-making process, contingent upon the situation. HCS assume different roles: an intervening/initiating advisory, a neutral advisory, or a recommendation-based role. HCS and NH strive for neutrality and caution in the advisory role, preferring to leave the decision-making within the family’s purview. HCS often sees its involvement as interference, though necessary when older adults’ care is at risk:

*“So*,* I’m always the neutral advisor. I’ll never take sides*,* but I always assess the current situation. But I do intervene when I see that there are simply dangerous situations.”* (HCS2_HCSmanager1).

NHs also reported being sought for advice regarding the decision of moving into a NH, yet they feel unable to provide advice due to their limited familiarity with the family and the caregiving situation:

*“It’s very difficult to advise because you don’t know the person who’s coming. I can’t really advise on this because I always say it’s up to the family to decide. […] Sometimes I have the feeling that I have to make this decision*,* can’t you do it for me? (laughs) But I can’t*,* and I don’t.”* (NH2_NHmanager1).

#### Pre-transition phase: suggestions for improvement

##### Early engagement with the future care situation

HPs from NHs and informal caregivers recommended early, collaborative conversations and timely information gathering for improving the transition to NH placement. *“The key is to start early and confront the issue*,* even if it’s unpleasant.”* (NH1_informalcaregiver2) emphasized an informal caregiver.

*“So*,* the best starting point is always when the resident agrees with their informal caregivers that they want to move. And then look together at the homes that are suitable for them. […] And yes*,* perhaps at a time when they are still physically able to settle in properly.”* (NH3_NHnurse2).

##### Independent counselling centre

A HP from a HCS and an informal caregiver expressed interest in an independent counselling centre that not only offers guidance but also takes on responsibilities for informal caregivers, such as assisting in NH searches and managing related arrangements. The advisory centre should be independent because, as stated by a relative, “*the health insurance company is only focused on saving money“* (NH5_informalcaregiver1).

The HP wished for a more hands-on approach from counselling centres, suggesting they should provide a place where informal caregivers could simply go and say,

*“This is our situation. We want to go to this or that nursing home. Please organize everything we need to do.”* (HCS5_HCSnurse1).

### Mid-transition phase

**Fig. 3:**
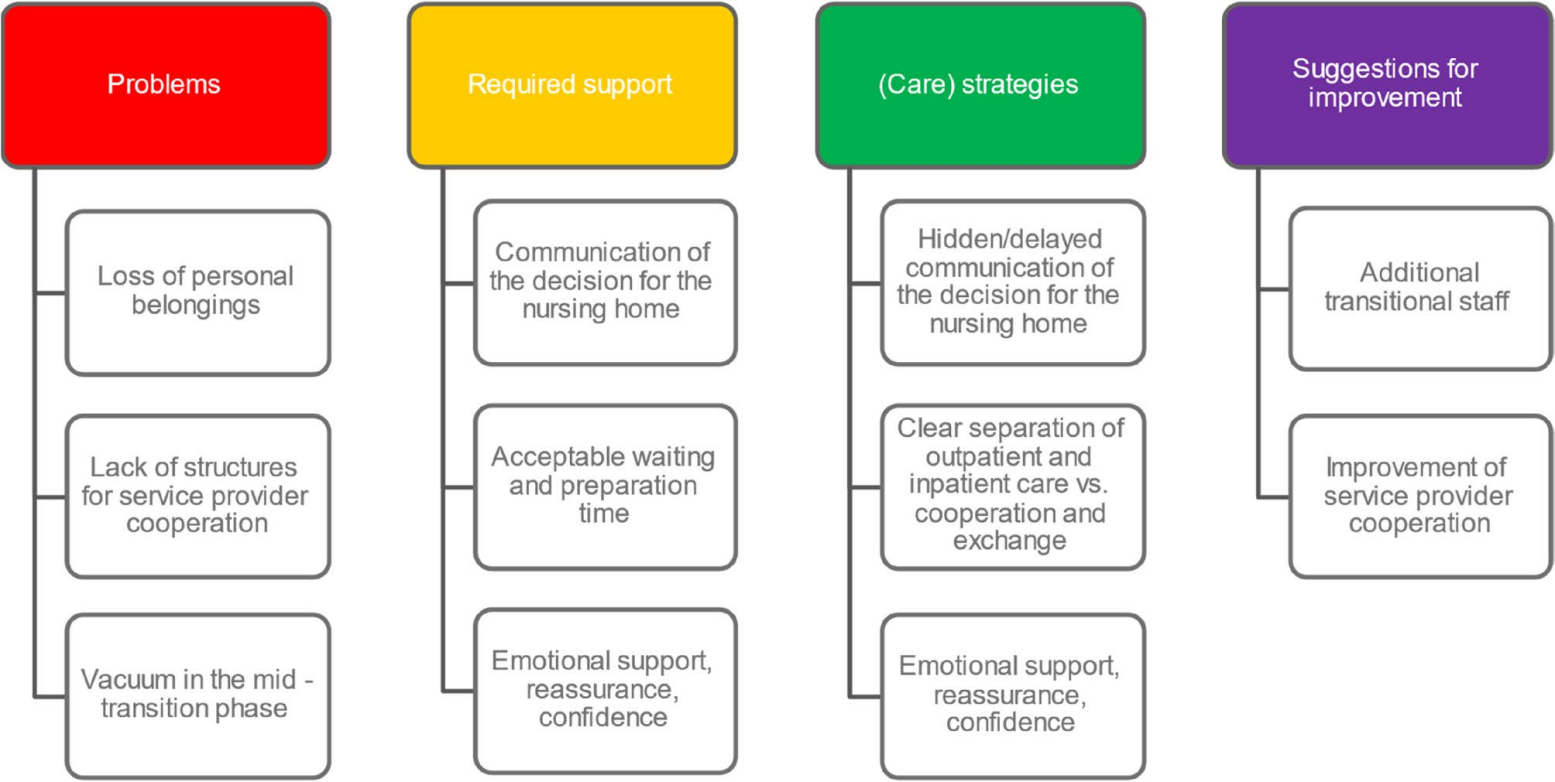
Overview of categories from the Mid-transition phase

Figure 3 provides an overview of the various categories of the mid-transition phase.

#### Mid-transition phase: Problems

##### Loss of personal belongings

HPs from NHs and residents noted challenges with leaving personal belongings and reduced living space when transitioning to NHs. Residents reported distressing experiences, sometimes unaware of their cherished items’ whereabouts. The loss of personal belongings was often linked to household clearances during the mid-transition phase. Transitions were perceived as rushed, leaving no time for calm contemplation on packing, as one resident stated:

*“I’m still very much at home in my head*,* right. And then it happened so quickly*,* blow by blow. […] The flat has been cleared*,* there are still a lot of things at home that I miss very much.”* (NH1_resident1).

Another resident also mentioned that in the rush, he forgot or didn’t even think to pack many of his belongings. Furthermore, he reported that not only was he never asked by his family what he wanted to take with him—his tablet containing all his memories was simply given away—but decisions were also made for him about what he was allowed to bring:

*“And they said*,* ‘That won’t all fit in the room. So*,* you don’t need to bring it with you.’ Although everything would have fit*,* like the pictures and everything else.”* (NH5_resident2).

Another resident mourned the separation from a beloved pet, now living with a former neighbour, expressing,

*“I do miss my cat*,* yes. But I had to give her up because I couldn’t take her with me.”* (NH4_resident1).

HPs from NHs are generally aware that many residents must part with personal belongings and pets due to limited space, facility restrictions or hurried transitions. However, they see no possibility of changing this circumstance.

##### Lack of structures for service provider Cooperation

A HCS manager reported insufficient structures for collaborations, such as lacking reimbursement for services within the context of the transition process, a lack of standards, data protection issues, and a high level of voluntary workload:

*“Yes*,* but the truth is*,* it’s not paid. When I’m describing a person with dementia*,* I sometimes sit for an hour. And the business hour simply costs money.“* (HCS2_HCSmanager1).

##### Vacuum in the Mid-transition phase

Informal caregivers reported a lack of emotional and organizational support from HPs during the mid-transition phase. Some caregivers did not use an HCS, while others received no advice despite having one. One caregiver noted that the only interaction with the HCS regarding the NH transition was the notification of the decision regarding their mother’s NH admission, with the HCS showing no surprise:

*“And the home care service was also aware of it; they were not surprised that we were placing my mother in a home because the living situation was so bad and she was so alone.”* (NH5_informalcargiver2).

Without the support of HPs, informal caregivers had to navigate this phase entirely on their own, as the caregiving relationship with the HCS ended, and they had not yet established a relationship with the NH. This frequently led to feelings of overwhelm, creating a vacuum during the mid-transition phase:

*“I think you’re totally left on your own when you’re in a situation like that.”* (NH5_informalcaregiver1). The moving day itself was also considered emotionally and organizationally challenging, as the informal caregiver further described:

*“There’s the emotional strain with my father*,* seeing and hoping that he really gets into my car. That we would really go there because I couldn’t have forced him. […] Packing all the stuff*,* trying to think of everything […].“* (NH5_informalcargiver1).

#### Mid-transition phase: Required support

##### Communication of the decision for the nursing home

HPs from NHs reported that informal caregivers wish for support in communicating the decision for NH placement. However, NHs often feel inadequately positioned to participate in communicating the decision, as they are unfamiliar with the incoming residents and their family dynamics, hence limiting their ability to provide advice:

*“The family really needs to sort that out. I actually say that because they know their mum*,* their partner or whatever the relationship is*,* best*,* yes. Once he’s there*,* it’s our job to manage it well and to make sure that it goes well. But bringing them [to the nursing home]*,* how to explain this*,* we can only give one bit of advice.”* (NH1_NHmanager1).

##### Acceptable waiting and Preparation time

HPs from NHs reported that family members and those in need of care require an acceptable waiting and preparation period, allowing them to choose an entry point that suits them individually:

*“So*,* some people say: ‘Thank God I got a place straight away. I’ll take it straight away.’ And some say: ‘Oh no*,* it’s not that bad now. I’ve just made an enquiry.‘*” (NH2_NHmanager1).

Informal caregivers also emphasized that due to the high demand for places, information regarding waiting list status would be greatly appreciated. Older adults in need of care require the waiting period to mentally prepare themselves for the impending admission. A resident praised that a room was reserved for him for a while so that he could take his time to think it over:

*“I thought*,* well*,* actually*,* I quite like it. And then when we got downstairs*,* the woman said*,* ‘Yes*,* I’ll hold the room for you for 14 days. If you like it*,* you can have the room.‘”* (NH5_resident2).

#### Mid-transition phase: (Care) strategies

##### Clear separation of inpatient and outpatient care vs. cooperation and exchange

There are varying approaches to cooperation between HCS and NHs. Most HPs describe a clear separation between outpatient and inpatient sectors, with some viewing exchange as unnecessary and primarily as a task for informal caregivers. However, some participants reported collaborations between NHs and HCS or GPs, typically limited to specific HPs with established connections and personal commitment. HCS reported that NHs are either uninterested in receiving information or they fear that they portray clients as challenging, potentially leading to the client being at a disadvantage in the NH:

*“Yes*,* because the nursing homes often don’t want to know. Or*,* as I said*,* they are biased when they find out something about the patients because something might slip out.“* (HCS1_HCSmanager1).

##### Hidden/delayed communication of the decision for the nursing home

HPs from NHs and informal caregivers reported that decisions about NH placement were often communicated in a hidden or delayed manner. Participants described that, although the decision for NH placement had already been made by the informal caregivers during the pre-transition phase, informal caregivers avoided direct confrontation with the older adult, thus delaying the notification until the mid-transition phase. They often employed hidden communication to persuade the older adult in need of care to move, often suggesting the admission as temporary, as one informal caregiver explained:

*“And so*,* we actually sold him [her husband in need of care] on the idea of going into short-term care. It wasn’t entirely fair*,* but*,* you know*,* the first month is considered short-term care. And then it seamlessly transitioned into full-time care.” (NH_5_informalcaregiver2)*.

As mentioned earlier, HPs from NHs stated that informal caregivers desire assistance from the NH when communicating the decision, which is initially withheld. Often, it’s only during the settling-in period that considerations arise about how the communication can be jointly managed:

*“Exactly*,* the informal caregivers really have to decide that [how to communicate the decision] for themselves. […] And during the familiarisation phase*,* if a resident is no longer doing well or something like that*,* then we also contact the informal caregivers. And say: ‘Hey*,* now we have to find a solution*,* how to tell him differently’*,* or something like that. But it really is a process.“* (NH1_NHmanager1).

#### Mid-transition phase: Suggestions for improvement

##### Additional transitional staff

A NH manager suggested additional transition personnel as an improvement, citing that she currently manages admissions alone alongside her responsibilities as a manager:

*“Clearly*,* more staff. […] Because if the nursing staff had to take part in the admission*,* it wouldn’t work.“* (NH2_NHmanager1).

##### Improvement of service provider Cooperation

A participating HCS suggested improving the collaboration between NH and HCS as a proposed enhancement. This could involve standardizing documentation, implementing a case manager role, and introducing a checklist.

*“Exactly*,* I think that would make a lot of sense if it were simply standardised. […] I have the case manager here [in the NH]. And I have my contact person here in the home care department.“* (HCS2_HCSmanager1).

### Post-transition phase

**Fig. 4:**
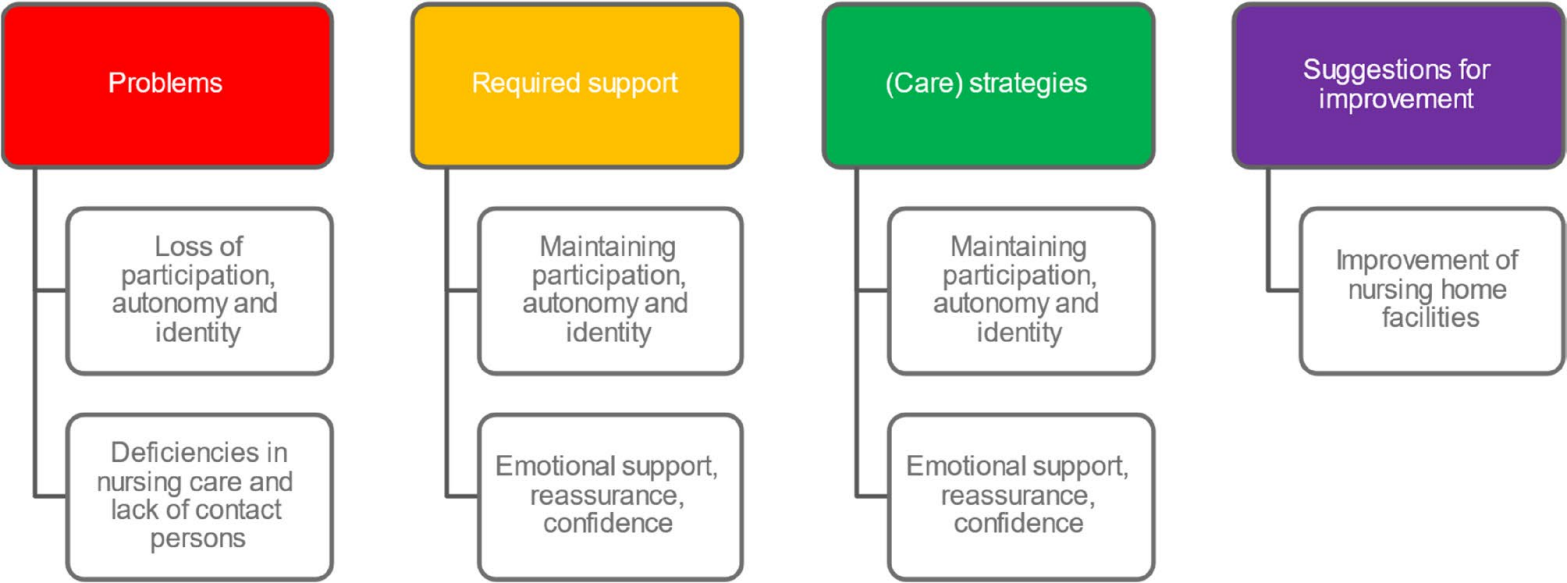
Overview of categories from the Post-transition phase

Figure 4 provides an overview of the various categories of the post-transition phase.

#### Post-transition phase: Problems

##### Loss of participation, autonomy, and identity

HPs from HCS and NHs and residents reported significant loss of participation, autonomy, and identity in older adults after moving to a NH. Residents expressed loss of continuity and personal connections due to facility regulations, reduced activities, or lack of visits. One resident likened the transition to experiencing his own death, as after death, all belongings are distributed to relatives and one’s identity fades away. For the resident, this loss of personal belongings was associated with a sense of worthlessness:

*“But on the other hand*,* it’s the little things that confirm that you’ve been deported. And that they are happy that they finally have nothing to do with things afterwards.”* (NH5_resident2).

Furthermore, the strong association of home with autonomy makes accepting the new living situation more difficult. A resident mentioned he couldn’t leave the NH alone due to facility rules, risking contract termination, as he lacked accompanying family members:

*“And they won’t let me out on my own because of the risk of accidents. […] ‘You can go out on your own*,* but then we’ll just have to cancel [the NH contract]’*,* she [the NH manager] said. […] Now I’m stuck in here and can’t get out.”* (NH4_resident1).

Other residents expressed dissatisfaction with the inactivity within the NH:

*“And basically*,* every activity is taken away from you.“* (NH5_resident2).

HPs acknowledged these structural restrictions, but they also noted that residents often surrender their autonomy and participation upon entering the NH, which they refer to as *“self-abandonment”*.

##### Deficiencies in nursing care and lack of contact persons

Informal caregivers expressed concerns about the adequacy of care for their loved ones, citing deficiencies in nursing care and a lack of consistent contact persons due to frequent staff turnover. One caregiver noted that this erodes trust in the provided care, prompting them to continually seek reassurance about the well-being of residents in the NH:

*“I always had to say*,* quite often: ‘You have to make sure my husband gets something to drink.’ Because he was hospitalised so often because he was really dehydrated.“* (NH2_informalcaregiver1).

#### Post-transition phase: Required support

##### Maintaining participation, autonomy, and identity

NH residents and informal caregivers wished for support in maintaining participation, autonomy, and identity. Caregivers sought assurance that their loved ones would be encouraged to make friendships and integrate well into the NH environment:

*“But when you see how well she is doing there*,* and she immediately has two or three women whom she calls friends. And yes*,* that is wonderful.”* (NH1_informalcaregiver1).

The residents themselves also reported the beneficial aspect of being supported and encouraged by the nursing team in engaging in meaningful activities:

*“We sometimes clean vegetables*,* peel potatoes*,* leeks*,* celery*,* whatever we need to chop*,* we do that. It’s an activity for us*,* yes*,* that’s fine*,* that’s what we do.“* (NH1_resident1).

Post-transition phase: (Care) strategies.

##### Maintaining participation, autonomy, and identity

HPs from NHs reported strategies aimed at preserving and fostering autonomy, continuity, identity, and personal connections among the residents. This includes gradual introduction into the community, involving informal caregivers, avoiding restraints, delegating tasks to the residents, as well as specialized house concepts. A NH manager reported their concept involves residents cooking with assistance and why this is important to many residents:

*“[…] At home they may have had food delivery services […]. And here a housewife can still do meaningful work*,* she is still needed.*“ (NH1_NHmanager1).

#### Post-transition phase: Suggestions for improvement

##### Improvement of nursing home facilities

As a suggestion for improvement, informal caregivers highlighted the need to enhance the NHs facilities, such as establishing a multipurpose room, providing more space in the rooms for personal belongings and improving meal variety. Other suggestions included tailoring activities for residents:

*“I would have thought it would have been nicer if it had been differentiated that those with mild to moderate dementia still had someone to communicate with […] And apart from that*,* it’s a drama in all nursing homes that the services are always very little tailored to male residents.“* (NH1_informalcaregiver2).

##### Emotional support, reassurance and confidence as a strategy and a required support across all phases

HPs from HCS and NHs cited emotional support as a strategy to support informal caregivers and residents. These involve instilling confidence, providing encouragement, and showing appreciation during the whole transition process. The assurance and validation that the decision for NH placement was the right choice was also deemed essential to overcome feelings of guilt, as one informal caregiver explained:

*“When you have to give your father away*,* because it always feels like giving him away […] you want someone to say: ‘It’s good.’ […] And the nursing home management really encouraged us that*,* from their point of view*,* the nursing home is not the end and deportation*,* but that it is still a life worth living.“* (NH1_informalcaregiver2).

HCS report that once the decision has been made, older adults in need of care need encouragement for the upcoming admission:

*“To speak well of things [the NH]. And really*,* yes*,* to give confidence. […] Encouragement on our part.“* (HCS5_HCSnurse1).

Residents also mentioned the importance of emotional support, such as connections with fellow residents or HPs. One resident praised the NH staff’s kindness for easing the transition:

*“Well*,* and then*,* I was actually here now. And they [NH staff] were all very friendly and very nice. They made the transition easy for me*,* actually.”* (NH5_resident2).

## Discussion

### Summary of main results

This study sheds light on care triad members’ experiences during the transition from home to NH, highlighting problems, strategies, needed support, and improvement suggestions. Our findings indicate the transition is frequently perceived as a downward journey. Despite desires for collaborative decision-making, family disagreements and decision postponements hinder progress. HPs tend to involve themselves late or not at all, resulting in little guidance or support. Decisions about NH placement lack transparency for older adults, who often perceive the process as rushed. Loss of personal belongings in mid-transition closely links to loss of identity, participation, and autonomy. Efforts by NHs to maintain these core self-aspects often fail. Emotional support is crucial but frequently lacking, leading to isolation and declines in participation and identity. Early engagement with future care arrangements may mitigate this negative cycle.

### Key differences in the experiences of care triad members

Most aspects were recognised by all groups, with key differences in perceptions of counselling: informal caregivers seek more guidance, while HCS and NHs often avoid active involvement in decision-making and communication, fearing they might cross professional boundaries or interfere in private family matters. Paradoxically, HPs from HCS also reported being confronted with finalised NH admission decisions without prior involvement, although they would have welcomed being included. At the same time, neither these HPs nor the informal caregivers proactively reached out to each other to discuss the care situation and a potential transition to a NH. This results in a mutual lack of communication and missed opportunities for guidance.

The gap of support and guidance during the mid-transition phase is primarily noted by informal caregivers, while HPs either remain unaware or attribute responsibility solely to either inpatient or outpatient care, leading to a shift and diffusion of accountability. Cooperation between HCS and NHs was perceived as heterogeneous: While the majority saw collaboration as unnecessary, a minority had established communication channels or expressed a desire for improvement. This indicates a clear separation of the inpatient and outpatient care, and the absence of shared standards for transitional processes.

### Research in context

Many issues related to the transition to NHs identified in this study were also identified in other research. These include the lack of alternatives due to complex health conditions or the overwhelming burden on caregivers [29], as well as stress during and after the transition for both informal caregivers and older adults in need of care [30, 31]. Additionally, diminished participation, autonomy and identity of older adults [32], and complexities in decision-making, including postponing the decision [4], further complicate the situation. This study highlights that despite the continuous need for support and guidance during the transition [8], it is neither provided in current practice, nor in transitional interventions.

#### Fragmentation and perceptual gaps in transitional care

Our study found that informal caregivers often feel overwhelmed by the tasks required before the admission, a finding supported by previous studies showing poor communication about older adults between healthcare services, leaving informal caregivers to manage all communication [32]. Our study findings suggest that this vacuum in the mid-transition phase originates from the previously mentioned fragmented care [33]. This leads to unclear accountability of HCS and NH, thereby placing the entire responsibility on informal caregivers. Discussions and workshops involving care triad members, conducted after the interviews were completed and analysed, revealed that the vacuum in the mid-transition phase does not seem to be apparent to HPs. Approaches that could close this gap, such as structured guidance, are not implemented as standard practice [17]. Importantly, while existing literature identifies gaps in continuity [17, 34], our findings highlight a perceptual misalignment: HPs view themselves as peripheral actors who do not feel responsible for the mid-transition phase and tend to shift responsibilities between services, whereas informal caregivers expect central coordination and guidance. Consequently, this critical period—when older adults in need of care and their informal caregivers require intensive guidance and support—is often overlooked. Moreover, while our data already suggested limited cooperation between NHs and HCS, indicative of a broader absence of binding standards or guidelines for transitional care, the full extent of this issue became particularly clear during workshop discussions with care triad members: Institutions revert to informal practices which are frequently inconsistent, even within the same organisation. As a result, responsibilities are unclear und needs from older peoples and their families frequently remain unmet.

#### Limitations of current transitional care interventions

Despite these structural and perceptual challenges in transitional care, interventions described in the international literature offer little guidance. Existing interventions tend to focus on emotional support and are typically provided by psychologists. This presents feasibility challenges within many countries’ healthcare systems. Furthermore, such supportive interventions primarily target the post-transition phase [18, 19], which is inadequate as unresolved issues in the preceding phases exacerbate the adjustment process [8] – a trend which was also observed in the current study. The temporal misalignment of interventions—focusing too late in the trajectory—may reflect a lack of a psychosocial approach: support is provided after institutionalization is “completed,” rather than during decision-making or relocation, when uncertainty and emotional distress are very high. Our findings further highlight a need to reframe the transition as a continuum of vulnerability requiring phased, context-sensitive support.

This lack of continuous support is especially concerning given the emotional burden associated with the transition. The decision-making and relocation process often proves painful for both informal caregivers and older adults in need of care, frequently leading to familial conflicts and feelings of guilt, while leaving the care recipients with a sense of abandonment [5, 12]. Additionally, the loss of personal belongings, which are integral for maintaining a sense of connection [12] was often accompanied by feelings of abandonment, grief, and worthlessness. From the perspective of older adults in need of care, a NH transition represents a biographical disruption—an involuntary step into dependency. These insights highlight the need for transitional care that supports not only functional needs but also preserves personal identity. The emotional strain caused by feelings of guilt, loss, and biographical disruption could potentially be alleviated through early, collaborative discussions involving HPs. Therefore, it is essential for HPs to comprehend the needs of older adults in need of care and their informal caregivers and to employ structured approaches to guide and motivate them in their shared decision-making process throughout the entire transition period [8, 11, 35–37]. This, however, is not prioritized in transitional care interventions at this point [35].

### Recommendations for further research and practice

HPs should be sensitised to recognise which phase of the transition individuals are currently in. This awareness helps them to assess the specific needs and challenges that arise at each phase. Understanding the transition phase can also clarify why decisions about NH transitions may be delayed, and enables HPs to provide timely emotional and organisational support tailored to individuals’ evolving needs throughout the process. HCS need to be made aware of their significance in the transitions from home to NHs, as they currently tend to withdraw from decision-making processes. Moreover, there is potential in systematically preparing future residents and their informal caregivers for the transition process early on, such as advising them to pack items of personal significance upon admission.

Specific suggestions drawn from our findings include fostering early collaborative engagement and accessible, independent guidance. In the mid-transition phase, implementing dedicated transitional staff and strengthening coordination between care providers may support a smoother transition. Finally adapting the NH environment and activities to residents’ individual needs, facilitating their adaptation in the post-transition phase.

Overall, future intervention strategies should adopt a proactive approach, encouraging individuals to consider their future care early and facilitating constructive discussions within their families. Interventions should start as early as possible and provide continuous support throughout all phases of the transition. It is also crucial to develop approaches that can be easily integrated into the existing workflows amidst the current healthcare crisis in Germany and many other countries.

### Strengths and limitations

This study has several strengths. First, it addresses a significant research gap by exploring diverse experiences across all phases of the transition process within members of the care triad, offering a more nuanced insight where similar comprehensive studies are limited. Second, a theoretical model guided the formulation of interview questions, providing a clear framework for data collection. The study successfully identified implications essential for developing interventions to enhance the transition from home to NHs.

Several limitations should be noted. The study utilized convenience sampling, which may affect representativeness. Given the ongoing challenges in many HCS and NHs due to COVID-19, this approach was justified but should be considered when interpreting results. Recruiting individuals through gatekeepers may have limited our sample, potentially leading to the inclusion of more cooperative individuals. However, this was required by the ethics committee to ensure that only individuals assessed as being emotionally stable were included, to minimise the risk of psychological distress. Nonetheless, we managed to include participants with diverse transition experiences. Another limitation of our study lies in its descriptive approach primarily aiming to inform the development of an intervention. As such, our study prioritized breadth and applicability over further developing a theory on transitions. Moreover, as residents and informal caregivers were only interviewed after the transition had taken place, all information about the transition process was collected retrospectively. This means that participants were not followed during the transition process and their reports may be subject to recall bias. However, we aimed to reduce this risk by including only residents who had been living in the nursing home for at least six weeks but no longer than six months, to ensure that the transition experience was still relatively recent. Finally, our study did not include complete care triads, but rather analysed individuals belonging to different triads. Future studies should aim to recruit entire triads to enable an in-depth exploration of the dynamics and mutual influences among their members.

## Conclusions

This study provides key insights into the transition from home to NHs based on the experiences of care triad members in the German health care system. Transitions are often delayed due to postponed decisions, family disagreements, and insufficient support from HPs. Informal caregivers frequently bear the full responsibility, leaving them feeling overwhelmed and lacking comprehensive support. As a result, transitions are often rushed, with older adults commonly losing autonomy, participation, and identity. Proactive engagement in planning transitions and future care situations, along with counselling from HPs could help prevent these negative outcomes and improve the experience for all involved.

The insights from this study, along with findings from a scoping review and expert workshops, lay the groundwork for developing a comprehensive intervention strategy focusing on participation, autonomy, and support. The intervention will be piloted in a study that includes a thorough process evaluation.

## Supplementary Information

Supplementary Material 1.

Supplementary Material 2.

Supplementary Material 3.

## Data Availability

All the data are available from the corresponding author on reasonable request.

## Abbreviations

HCS: Home Care Service
HP: Health Professional
GP: General Practitioner
TRANSCIT: TRANsition, Support, Communication, Information, and Time
NH: Nursing Home
MRC: UK Medical Research Council
SRQR: Standards for Reporting Qualitative Research
