# Supplementary material for: Experiences of members of the care triad on transitioning from home to a nursing home: a qualitative study
Source: BMC Geriatr. 2025 Nov 4;25:844. doi: 10.1186/s12877-025-06586-1 (PMC12584320; doi:10.1186/s12877-025-06586-1)
Supplement: Supplementary file 1 — Supplementary Material 1. [file 12877_2025_6586_MOESM1_ESM.docx]

Supplementary file 1: Interview guides HPs from HCS, informal caregivers and NH residents

Interview guide HPs from HCS

| Part I: Pre-Transition Phase: This section of the interview focuses on the decision-making period, specifically just before the decision to move into a nursing home is made. |
| --- |
| 1. How do you notice that patients or their informal caregivers are starting to consider the move into a nursing home?    1. Key Elements: Information/Support  To what extent are you aware of how the decision to move into a nursing home is made by those affected and their families?    2. Key Elements: Support/Communication/Time/Information  What support do those affected and their families receive to help them make the decision to move into a nursing home?    3. Key Elements: Communication/Time In your experience, what emotions do the affected individuals and their families experience during the decision-making phase? |
| Part II Mid-Transition Phase: Now, let's focus on the time period after the decision for the nursing home has been made up until the time of move-in. |
| 1. What happens after the decision for the nursing home?    1. Key Elements: Support/Information/Communication/Time   How are the families supported in the preparations for the move to the nursing home?   - 1. Key Elements: Communication/Time   In your experience, what feelings do the future residents and their families have during the transition phase until moving into the nursing home? |
| Part III Post-transition Phase : Now, I'd like to discuss the time after the move-in. |
| 1. How do things proceed in the period after the move? |
| Part IV Conclusion: Looking back at the entire transition process from decision to move into the nursing home, what is working well in your home care nursing service, and what could be improved?  Open-ended closing question: Is there anything else you would like to add that I haven't asked about? |

Interview guide informal caregivers

| Part I: Pre-Transition Phase: First, I would like to learn more about the period just before the decision to move into a nursing home was made.. |
| --- |
| 1. What can you remember particularly well when you think back to this time? Is there a specific situation that stands out in your memory?    1. Key Elements: Information/Support/Communication How was the decision to move into the nursing home made?    2. Key Elements: Information/Support/Communication  What information did you receive regarding a possible move into a nursing home?    3. Key Elements: Communication/Time How did you feel emotionally during the time when the decision to move into the nursing home was made? What support did you need during this time? What support did you actually receive? |
| Part II Mid-Transition Phase: Now, let's focus on the time period after the decision for the nursing home has been made up until the time of move-in. |
| 1. How did things proceed after the decision to move into the nursing home was made?    1. Key Elements: Information/Communication/Support   How did you feel emotionally while preparing for the move? To what extent were you supported in preparing for the move into the nursing home?   - 1. Key Elements: Communication   How did you feel emotionally during the actual move-in? |
| Part III Post-transition Phase : Now, I'd like to discuss the time after the move-in. |
| 1. How do things proceed in the period after the move?    1. Key Elements: Communication/Time   How would you describe your relationship with the health professionals from nursing home?   - 1. Key Elements: Support/Information/Time   To what extent are you able to participate in the daily life of your loved one in the nursing home?   - 1. Key Elements: Support/Time   In your assessment, how well has your loved one actually settled into the nursing home? How integrated do you feel they are there? |
| Part IV Conclusion: Looking back at the entire transition process from decision to move into the nursing home, what is working well, and what could be improved?  Open-ended closing question: Is there anything else you would like to add that I haven't asked about? |

Interview guide NH residents

| Ice-Breaker: First, I would like to talk a little bit about your daily live. Would you mind sharing a bit about how your day has been going so far? |
| --- |
| - 1. It hasn't been long since you moved into the nursing home here. I'm particularly interested in the time before and after the move. Let's start with the moment when the decision was made to move into the nursing home. Then, tell me everything you can remember. We can take our time – every detail is important to me.   2. During the decision-making period and the process of settling into the nursing home, was there anything that particularly helped you or made you feel good?   3. What was difficult or burdensome for you during this time? What kind of help did you need? |
| Part IV Conclusion: What aspects of your life here in the nursing home do you particularly enjoy? Could you share a situation or example? Alternatively: What factors have contributed significantly to your ability to feel at home or settle in here? Alternatively: If you haven't quite settled in yet, what do you think could help you do so? Open-ended closing question: Is there anything else you would like to add that I haven't asked about? |
