# Supplementary material for: Experiences of members of the care triad on transitioning from home to a nursing home: a qualitative study
Source: BMC Geriatr. 2025 Nov 4;25:844. doi: 10.1186/s12877-025-06586-1 (PMC12584320; doi:10.1186/s12877-025-06586-1)
Supplement: Supplementary file 3 — Supplementary Material 3. [file 12877_2025_6586_MOESM3_ESM.docx]

Supplementary file 2: Overview categories and contribution of the different participant groups

| **Phase** | **Subcategories** | **HPs from HCS** | **HPs from NH** | **Informal caregivers** | **Older adults** |
| --- | --- | --- | --- | --- | --- |
| **Pre-transition phase: problems** | Inevitability / Lack of alternatives | X | X | X | X |
|  | Complexities in decision-making processes | X | X | X | X |
| **Pre-transition Phase:**  **required support** | Shared & constructive decision | X | X | X |  |
|  | NH recommendations and information about the future home | X | X | X | X |
| **Pre-transition phase:**  **(care) strategies** | Early engagement with the future care situation | X | X | X | X |
|  | Situational involvement in the decision-making process | X | X |  |  |
| **Pre-transition phase: suggestions for improvement** | Early engagement with the future care situation |  | X | X |  |
|  | Independent counselling centre | X |  | X |  |
| **Mid-transition phase: problems** | Loss of personal belongings |  | X |  | X |
|  | Lack of structures for service provider cooperation | X |  |  |  |
|  | Vacuum in the mid-transition phase |  |  | X |  |
| **Mid-transition phase: required support** | Communication of the decision of the NH |  | X |  |  |
|  | Acceptable waiting and preparation time |  | X | X | X |
| **Mid-transition phase: (care) strategies** | Clear separation of the inpatient and outpatient care vs. cooperation and exchange | X | X |  |  |
|  | Hidden/ delayed communication of the decision for the NH |  | X | X |  |
| **Mid-transition phase: suggestions for improvement** | Additional transitional staff |  | X |  |  |
|  | Improvement of service provider cooperation | X |  |  |  |
| **Post-transition phase: problems** | Loss of participation, autonomy and identity | X | X |  | X |
|  | Deficiencies in nursing care and lack of contact persons |  |  | X |  |
| **Post-transition phase: required support** | Maintaining participation, autonomy and identity |  |  | X | X |
| **Post-transition phase:**  **(care) strategies** | Maintaining participation, autonomy and identity |  | X |  |  |
| **Post-transition phase: Suggestions for improvement** | Improvement of the NH facilities |  |  | X |  |
| **All phases: required support/ (care) strategies** | Emotional support, reassurance, confidence | X | X | X | X |
| *NH = nursing home, HP = health professional, HCS = home care nursing service* | | | | | |
